# Cortisol as a Biomarker for Stress During the Assessment and Treatment of Destructive Behavior

**DOI:** 10.3390/bs15040475

**Published:** 2025-04-06

**Authors:** Sean W. Smith, Paul R. Johnson, William E. Sullivan, Courtney R. Mauzy, Beatriz E. Arroyo Antúnez, Andrew R. Craig, Alexandra R. Howard, Thanh Nguyen, Chelsea Hoffman, Samhitha Adavikolanu, Henry S. Roane

**Affiliations:** 1 Biobehavioral Health Unit, 655 Madison St., Syracuse, NY 13210, USAnguyelam@upstate.edu (T.N.); roaneh@upstate.edu (H.S.R.); 2 Department of Pediatrics, SUNY Upstate Medical University, 750 East Adams St., Syracuse, NY 13210, USAarroyoab@upstate.edu (B.E.A.A.); adavikos@upstate.edu (S.A.); 3 Department of Clinical Laboratory Science, SUNY Upstate Medical University, 766 Irving Avenue, Syracuse, NY 13210, USA

**Keywords:** destructive behavior, stress, cortisol, biomarker, assessment, treatment

## Abstract

Behavior-analytic treatments successfully reduce individuals’ destructive behavior (e.g., self-injurious behavior, aggression, property destruction, disruption); however, there is limited research evaluating these treatments’ effects on individuals’ physiological stress responses, even though stress can have significant negative impacts on quality of life. Research from other fields has demonstrated that salivary cortisol concentration is a valid biomarker for stress, so researchers could potentially use this biomarker to assess the stress of individuals with limited communication repertoires who also engage in destructive behavior. The purpose of this research was to assess changes in salivary cortisol concentrations as a biomarker for stress with two participants to evaluate whether conditions that evoke destructive behavior induce stress relative to conditions that do not evoke destructive behavior. For one participant, salivary cortisol concentrations tended to increase following exposures to stimuli that evoked destructive behavior compared to conditions that did not evoke destructive behavior. The other participant had elevated salivary cortisol concentrations across all conditions. Salivary cortisol may be a useful biomarker for evaluating physiological stress as an outcome measure during research on the assessment and treatment of destructive behavior.

## 1. Introduction

Using the principles of behavior analysis to assess and treat the destructive behavior (e.g., self-injurious behavior, aggression, property destruction, disruption) of individuals with neurodevelopmental differences (e.g., autism spectrum disorder [ASD], intellectual/developmental disability [IDD]) is highly effective (Campbell, 2003; Heyvaert et al., 2014; Kurtz et al., 2011; Roane et al., 2016). A behavior-analytic approach often begins with a functional analysis (FA; Iwata et al., 1982, 1994; see Melanson and Fahmie, 2023, for review), which clinicians use to identify the specific reasons a particular individual engages in destructive behavior. Research has identified that about 83% of individuals who engage in destructive behavior do so in response to specific stimulus changes in their environment (i.e., they have socially mediated functions of their behavior; Melanson & Fahmie, 2023). For example, this research shows that destructive behavior occurs specifically in response to events like losing access to a preferred toy (e.g., iPad), losing access to attention from preferred people (e.g., parents, behavior technicians), or being instructed to engage in nonpreferred tasks (e.g., academic instruction, showering). When clinicians identify that destructive behavior occurs in response to specific changes in the environment for individuals with limited communication repertoires, treatments based on functional communication training (FCT) result in greater than 80 percent reductions in destructive behaviors in over 90 percent of treatment applications (Ghaemmaghami et al., 2021; Greer et al., 2016).

Although treatments like FCT are highly successful at reducing destructive behavior, there is limited research evaluating whether stimuli that occasion destructive behavior in this population induce stress (van der Linden et al., 2022). Similarly, there is limited research evaluating whether treatments can successfully reduce an individual’s stress when they experience the environmental stimuli that previously occasioned their destructive behavior. This is concerning because large-scale reviews have identified that individuals diagnosed with neurodevelopmental differences like ASD may be more susceptible to experiencing clinically significant hypothalamic–pituitary–adrenal axis dysregulation and increased levels of stress relative to neurotypical peers (Taylor & Corbett, 2014; van der Linden et al., 2022), which may also contribute to increased destructive behavior (for example, see Foley and Baz (2021), for discussion). This stress can have widespread effects on an individual’s ability to function independently and their overall quality of life (e.g., Bishop-Fitzpatrick et al., 2015; McQuaid et al., 2022). Thus, it is necessary to evaluate whether the environmental stimuli that cause destructive behavior also induce stress and whether the current standard of care for the treatment of destructive behavior can reduce patients’ biological response to clinically relevant environmental stressors.

Importantly, many individuals who engage in destructive behavior also have limited communication repertoires and are unable to communicate their basic needs or emotional states (Neuhaus et al., 2022). These communication deficits present a significant barrier to assessing stress for this population because stress is often assessed via self-report in communication modalities that these individuals are unable to use effectively. Given the profound and widespread effects of stress on quality of life, there is a need for researchers to develop an alternative way to assess stress in this population.

Developing a way to assess stress for individuals who both engage in destructive behavior and have limited communication repertoires could have significant clinical implications. These individuals engage in frequent destructive behavior because they are exposed to environmental stimuli that induce these behaviors multiple times daily (Melanson and Fahmie, 2023). Although treatments may reduce destructive behavior, it is unclear whether these treatments reduce stress. Thus, clinicians are unable to ascertain whether these individuals may continue to experience acute stressors frequently throughout the day, even after receiving “successful” treatment for their destructive behavior. Furthermore, professionals (e.g., researchers, clinicians) and nonprofessionals have both suggested that behavior-analytic assessment and treatment procedures may be traumatic (e.g., Kupferstein, 2018; Rozsa, 2025; cf. Rajaraman et al., 2022, and Kranak & Briggs, 2025); however, it is difficult to objectively quantify the extent to which this is true for individuals with limited communication repertoires. Identifying a novel way to objectively quantify stress would help researchers begin to assess and address these concerns systematically.

Continued exposure to stress can lead to decreased independent functioning and quality of life (e.g., Bishop-Fitzpatrick et al., 2015; McQuaid et al., 2022). Developing a way to accurately assess these individuals’ stress would provide researchers with a new measure for determining the social validity of the assessment and treatment process and the ultimate successfulness of clinical treatments. It would allow researchers to (a) determine the extent to which certain assessments and treatments induce or reduce stress and (b) refine assessments and treatments until they successfully minimize both destructive behavior and stress. Such assessment and treatment refinements could ultimately improve the overall quality of life of these individuals by reducing their stress in addition to reducing their destructive behavior.

Research has established that the concentration of cortisol in a person’s saliva is a reliable biomarker for stress (Giacomello et al., 2020; Hellhammer et al., 2009), so it may be possible to use salivary cortisol concentrations as an objective measure of stress for people who are not able to communicate about their stress directly. More specifically, extensive research has demonstrated that exposing people to acute stressors will reliably increase their salivary cortisol concentrations, and these increased levels of cortisol correlate both with self-reported measures of stress and many other physiological indicators of stress (e.g., Giacomello et al., 2020; Kirschbaum et al., 1993; Schlotz et al., 2008).

To use salivary cortisol as a biomarker for stress, it is important to consider at least two general findings. First, baseline salivary cortisol concentrations can vary greatly across days and participants (Danese et al., 2024). Thus, when using salivary cortisol as a biomarker for stress during exposure to acute stressors, it is necessary to conduct within-subject comparisons of salivary cortisol concentrations before and after exposure to the stressor. Second, there tends to be a delay before salivary cortisol concentrations increase after exposure to an acute stressor. Specifically, salivary cortisol concentrations tend to peak around 20−30 min after the stressor’s onset and then slowly decrease back to baseline levels about 90 min after the stimulus (Giacomello et al., 2020; Liu et al., 2017; Schlotz et al., 2008; Wolfram et al., 2013).

Corbett et al. (2006) conducted an experiment that serves as good example of how to use salivary cortisol concentration as a biomarker for stress. In this experiment, Corbett et al. evaluated changes in salivary cortisol concentrations for participants with and without diagnoses of ASD before and after exposure to a mock MRI scan procedure (i.e., a potentially stressful event). First, Corbett et al. collected participants’ saliva immediately upon arrival to the experimental context. Next, they exposed participants to the 20 min mock MRI scanning procedure. Finally, they collected participants’ saliva 20, 40, and 120 min after exposure to the mock MRI scan. Participants diagnosed with ASD had increased salivary cortisol concentrations shortly after exposure to the mock MRI scan (i.e., at 20 and 40 min after the mock scan) compared to their cortisol concentrations in the first saliva sample collected during the appointment (i.e., prior to the mock scan). On the other hand, participants without ASD did not have increased cortisol concentrations after the mock scan. These results suggest that the mock MRI scan produced a physiological stress response for participants with ASD, but not for participants without ASD.

Extensive research has used similar methods to evaluate salivary cortisol with individuals with ASD (see van der Linden et al., 2022, for review). In fact, some of this research has demonstrated that individuals with ASD have different (a) diurnal patterns of cortisol, (b) baseline cortisol concentrations, and (c) increased cortisol reactivity in response to acute stressors (van der Linden et al., 2022).

Although research has used salivary cortisol as a biomarker for stress for individuals with ASD, there has been limited research evaluating cortisol in relation to specific environmental events that might (a) occasion destructive behavior or (b) be particularly stressful for individuals with ASD (e.g., waiting for preferred things/events, completing nonpreferred instructions, being told “no”; van der Linden et al., 2022). Notably, these events, which are socially significant for this population but have not yet been evaluated, tend to occur frequently in their daily routines and are the same environmental events commonly found to evoke destructive behavior during FAs (Melanson & Fahmie, 2023). This highlights a need for evaluating whether the stimuli that evoke destructive behavior also induce stress and whether stress reduces when these destructive behaviors are treated.

Toward this goal, we evaluatedthe salivary cortisol concentrations of two patients diagnosed with ASD during the assessment and initial treatment of their destructive behavior. Specifically, we evaluated their salivary cortisol concentrations during an FA and a treatment evaluation of FCT, both conducted with a pairwise design for both participants. This allowed us to compare their salivary cortisol concentrations before and after potentially high- and low-stress conditions that did and did not evoke destructive behavior, respectively. In this way, we evaluated whether their salivary cortisol concentrations tended to fluctuate in relation to (a) the presentation of specific environmental stimuli and (b) destructive behavior. We hypothesized that presenting specific environmental stimuli that evoked destructive behavior would cause increases in their salivary cortisol concentrations and that the magnitude of the increase in their salivary cortisol concentration would be related to the rate of destructive behavior we observed in the presence of the environmental stimuli.

The more general purpose of this evaluation was to determine the feasibility and efficacy of using salivary cortisol concentrations as a biomarker for stress for patients diagnosed with ASD and/or IDD receiving services for the assessment and treatment of destructive behavior who may not be able to communicate about their stress more directly. This type of research may also help establish a methodology for evaluating biological mechanisms associated with behaviors in a way that can facilitate collaboration between behavioral and biological sciences and allow researchers to develop treatments to target stress as an outcome for this population.

## 2. Methods

### 2.1. Setting and Participants

Participants were recruited from patients receiving services at a university-affiliated outpatient clinic designed for the assessment and treatment of destructive behavior. To avoid coercion, participants were recruited by a researcher who was not a clinician responsible for providing clinical services to the patients. In the clinic, therapy rooms were equipped with padding on the walls, one-way observation windows, two-way intercoms, and digital video-monitoring and -recording equipment. All staff in this program received specialized training on safely managing destructive behavior (Bernstein et al., 2024), wore protective equipment as necessary, and had continuous access to doctoral-level clinicians to ensure safety throughout all assessment and treatment procedures.

Patients received clinical services during 1 h appointments conducted 5 days a week, and all research activities with our participants occurred within their regularly scheduled clinical appointments. After the study was initiated, all patients who began services were screened for inclusion based on the following criteria: (a) a routine clinical interview suggested their destructive behavior was not maintained by automatic reinforcement but occurred in response to manipulable environmental stimuli, (b) routine clinical observation corroborated the absence of destructive behavior maintained by automatic reinforcement, (c) their sex was male at birth, (d) they had the ability to attend afternoon appointments, and (e) there was no reported noncompliance with toothbrushing or dental procedures.

Although no patients were excluded based on these criteria for this experiment, we briefly outline the rationale for these criteria to facilitate potential replications of this research. We planned to exclude patients with behavior maintained by automatic reinforcement because these patients would not engage in destructive behavior in relation to specific, manipulable environmental stimuli. Excluding these participants would help ensure the selection of participants more likely to experience stress in relation to specific environmental stimuli that evoke their destructive behavior, which would increase the likelihood of detecting changes in salivary cortisol concentrations in relation to specific environmental stimuli. We planned to include only male participants in this initial study because prior research has suggested that salivary cortisol reactivity is greater for male participants compared to females (Liu et al., 2017), thereby enhancing the probability of detecting changes with a relatively small sample size in this initial research. Similarly, we planned to include patients who could attend afternoon appointments because, generally, salivary cortisol concentrations are highest in the morning and decrease throughout the day (Horrocks et al., 1990; Salimetrics, LLC, 2021), so the effects of exposure to acute stressful stimuli on salivary cortisol concentrations would be most easily detected later in the day (i.e., when concentrations would be lowest, permitting better detection of potential increases due to exposure to stressful stimuli). We planned to exclude patients with reported noncompliance with toothbrushing or dental procedures because this would limit the likelihood that participants would revoke their assent related to the saliva collection procedures, which involved inserting a swab into the participant’s mouth for 2 min multiple times per appointment.

Horace was an eight-year-old Caucasian boy diagnosed with ASD, IDD, and attention deficit hyperactivity disorder (ADHD). Horace was primarily referred for the assessment and treatment of his self-injurious behavior (SIB). Boris was a 12-year-old mixed-race (black, Caucasian) boy diagnosed with ASD, learning disability (not otherwise specified), expressive language disorder, auditory processing disorder, and ADHD; Boris had additional diagnoses of speech impairment, sensory processing difficulty, and developmental delay. Boris was primarily referred for the treatment of his aggressive and disruptive behavior. No other participants were recruited for this experiment.

We obtained informed consent from these participants’ caregivers. We also attempted to obtain assent from our participants. We described the procedures and their purpose and asked for permission to begin the procedures before starting the experiment. One participant did not respond affirmatively or negatively to this assent process. One participant responded affirmatively that he would participate. We would have considered resistance to the saliva collection procedure (e.g., turning the head side-to-side to avoid the swab, pushing the swab away) as removal of assent, but neither participant resisted the research-specific procedures. No adverse events occurred during this experiment. All research was conducted with the permission of our university’s institutional review board.

### 2.2. Materials

For both participants, an iPad was present and used as a positive reinforcer during sessions. Instructional materials were also present for both participants to set up negative reinforcement contingencies. For Horace, instructional materials were laminated sheets for the presentation of receptive identification tasks. For Boris, instructional materials were clothes for folding, toys to be put away in bins, and pieces of paper to be put in a recycling bin. A weighted chair and table were present throughout all sessions. During functional communication pretraining and treatment evaluation phases (described below), a communication device was present in the room for Horace, and functional communication response (FCR) cards were present for Boris to exchange with the session implementer to obtain functional reinforcers (i.e., iPad, break from instructions).

We collected and stored saliva samples using Salimetrics, LLC children’s swabs, storage tubes, and freezer storage boxes. We stored samples in a commercially available freezer rated for maintaining −20 °C, which maintains the integrity of salivary cortisol samples for at least 6 months (Salimetrics, LLC, 2021). We used an external thermometer to monitor the maximum and minimum daily temperatures, which remained below −20 °C throughout the time we stored the samples. All saliva samples were analyzed within 6 months of being collected. Samples were analyzed by our clinical laboratory scientist (second author) using a Salimetrics, LLC Enzyme Immunoassay test kit run on a BioTek Synergy LX Multimode Reader (Agilent, Santa Clara, CA, USA). Briefly, this method is based on the “sandwich principle”, in which a capture antibody specific to the biomarker of interest (cortisol) is pre-coated by the manufacturer onto a 96-well microplate. Thawed sample tubes were centrifuged to obtain saliva from the tube and subsequently added to the microplate wells in duplicate, according to the manufacturer’s instructions. Each plate used to analyze participant samples also contained a set of six quantitative standards (with known cortisol concentrations) to generate a calibration curve, along with one low-level and one high-level quality control samples to ensure the cortisol results were within the expected concentration ranges to validate the test run. All calibration standards and controls were run in duplicate, and these materials were provided by the manufacturer as part of the salivary cortisol test kit. Saliva samples from participants were thawed for analysis and were batched and run on the same day to ensure quality test results. After the addition of standards, controls, and participant saliva samples, an enzyme conjugated to a detection antibody was added to each well; the plate was then mixed on a rotator for 5 min, followed by incubation at room temperature (20–23 °C) for one hour to allow the specific binding of the cortisol present in the samples to the capture antibody and enzyme-conjugated detection antibody. A series of wash steps were subsequently performed to remove any non-specific binding to the plate wells. In the final steps, a substrate solution chemical (included with the test kit) was added to the wells to allow for the enzyme conjugate to convert the reagent substrate to a colored product. After a 25 min incubation period, a stop solution was added to all wells to achieve the final absorbance for measurement. The light absorbance measurements of all wells were analyzed on the BioTek plate reader with the primary wavelength set at 450 nanometers (nm) and with a secondary filter correction of 491 nm. Absorbance values were converted to concentration units (μg/dL, mcg/dL) based on the calibration curve generated with the standard solutions and mathematically interpreted by the plate reader using the manufacturer’s recommendation of a four-parameter non-linear regression curve fit.

### 2.3. Dependent Variables and Response Measurement

#### 2.3.1. Destructive Behavior

Trained data collectors recorded data on destructive behavior on laptop computers while observing sessions from behind a one-way observation window using BDataPro software (Bullock et al., 2017). We developed individualized definitions for each participant’s destructive behavior based on (a) caregivers’ descriptions of each patient’s destructive behaviors and (b) clinicians’ descriptions of the patients’ behavior during clinical programming that occurred prior to the research procedures.

For Horace, we collected frequency data on his SIB, defined as successful or blocked attempts of face-slapping (forceful contact of an open hand with the face), head-banging (forceful contact of the head with a stationary object), head-hitting (forceful contact of a closed hand or knee with any part of the head), self-scratching, neck-punching (forceful contact of the hand with any part of the neck), and self-pinching. We also collected data on the duration of Horace’s negative vocalizations, defined as any instance of screaming, whining, crying (with or without tears), or moaning in the absence of positive affect (i.e., smiling, laughing) with a 3 s onset/offset criterion. Horace’s negative vocalizations were not a treatment target, so these responses did not produce consequences during this experiment.

For Boris, we collected frequency data on his disruptive behavior, defined as successful or blocked attempts of throwing objects a foot or greater with force (excluding during appropriate toy play), kicking or hitting objects from a distance of 6 inches or greater with force, and overturning furniture. Additionally, we defined aggressive behavior as hitting, punching, kicking, pushing, and grabbing other people, but no aggressive behaviors occurred during our experiment. We also collected data on the frequency of Horace’s negative statements, which were short, discrete occurrences and were defined as instances of swearing or cursing.

We converted frequency data to rates by dividing the total count of the behavior by the number of minutes within the observation interval. We converted durations to a percentage of the observation interval by dividing the duration of the behavior by the entire duration of the observation interval and multiplying the quotient by 100.

#### 2.3.2. Salivary Cortisol Concentration

We collected participants’ saliva twice per 1 h appointment, once at the beginning of the appointment and again at the end of the appointment. Appointments were 2 pm–3 pm for Horace and 3:30 pm–4:30 pm for Boris. Consistent with other research using salivary cortisol as a biomarker for stress, the first collection of the day served as a baseline measure of the participants’ cortisol and a point of comparison for how much the cortisol concentration changed by the end of the appointment. This is necessary because salivary cortisol concentrations can vary from day to day based on several variables (e.g., diet, sleep, exposure to stress earlier in the day; Giacomello et al., 2020), so it is necessary to account for baseline concentrations to determine how much salivary cortisol may increase due to exposure to specific environmental stimuli.

To collect participants’ saliva, we followed the instructions provided by Salimetrics, LLC (n.d.). With gloved hands, we removed the swab from the package and placed one end of the swab in the participant’s mouth, either under their tongue or in between their lower gums and cheek, where saliva tends to pool. We provided both participants with synchronous reinforcement while they kept the swab in the appropriate position: participants were provided access to an iPad as long as the swab remained in place. The collection procedure ended after the swab had remained in an appropriate location for a total of 2 min. These 2 min of total collection time did not need to be consecutive, so breaks were permitted, but neither participant required more than one break per collection, nor did any break last for more than 5 s. After the saliva was collected, the swab was immediately placed in the storage container; labeled with the participant number, date, and time; and placed in a storage box in the freezer.

When both participants completed participation, we transferred the frozen samples to our on-campus laboratory for analysis. In the lab, the saliva samples were thawed and analyzed for cortisol concentrations using a Salimetrics, LLC 96-well Enzyme Immunoassay test kit run on a BioTek Synergy LX Multimode Reader. We analyzed each sample in duplicate and calculated the average concentration across these two readings to determine the final salivary cortisol concentration for a given sample, measured in micrograms per deciliter (μg/dL).

As noted earlier, it is necessary to account for baseline cortisol concentrations to determine how exposure to different environmental stimuli may cause changes in salivary cortisol concentrations. For this reason, we primarily report data in terms of changes in salivary cortisol concentrations in relation to (a) different environmental stimuli and (b) rates of destructive behavior. To calculate changes in salivary cortisol concentrations, we subtracted the cortisol concentration obtained from the sample collected at the beginning of an appointment from the concentration obtained from the sample collected at the end of the same appointment.

### 2.4. Interobserver Agreement and Procedural Fidelity

We video-recorded sessions, and an independent observer collected reliability and procedural fidelity data with BDataPro software (Bullock et al., 2017) on laptop computers as they watched the video recordings of the research sessions. For frequency data, we calculated interobserver agreement (IOA) by dividing each session into 10 s intervals, counting the number of intervals where the primary and reliability data collectors recorded the same number of responses, dividing this number by the total number of intervals in the session, and multiplying it by 100%. For duration data, we calculated IOA by dividing each session into 10 s intervals, dividing the lower duration recorded by the higher duration recorded within each interval, and calculating the average of this value across all intervals within the session.

For Horace, we calculated IOA for 93.3% of all sessions and obtained an average IOA value of 99.3% for SIB (range: 88.9–100%) and 94.6% for negative vocalizations (range: 70.5–100%). For Boris, we calculated IOA for 72.9% of all sessions and obtained an average IOA value of 99.6% for disruptive behavior (range: 95.6–100%) and 99.8% for negative statements (range: 95.6–100%).

To assess procedural fidelity, secondary data collectors recorded the frequency of omission and commission errors within each session. An omission error was defined as any instance of an implementer failing to execute a programmed antecedent or consequence within 5 s of when it was supposed to be performed. A commission error was defined as any instance of an implementer executing an unprogrammed antecedent or consequence.

For Horace, we collected procedural fidelity data for 100% of sessions. There were no omission errors during the experiment and an average of 0.07 commission errors per session (i.e., two commission errors throughout the experiment).

For Boris, we collected procedural fidelity data for 100% of sessions. Notably, Boris’ caregiver implemented all sessions. There was an average of 0.03 omission errors per session (i.e., one omission error throughout the experiment) and an average of 0.19 commission errors per session (i.e., four commission errors throughout the experiment).

### 2.5. Experimental Design

We analyzed rates of destructive behavior during an FA, functional communication pretraining, and treatment evaluation. The FA and treatment evaluation were conducted with a pairwise design.

### 2.6. Procedures

#### 2.6.1. General Procedures: Appointment Structure

**Figure 1:**
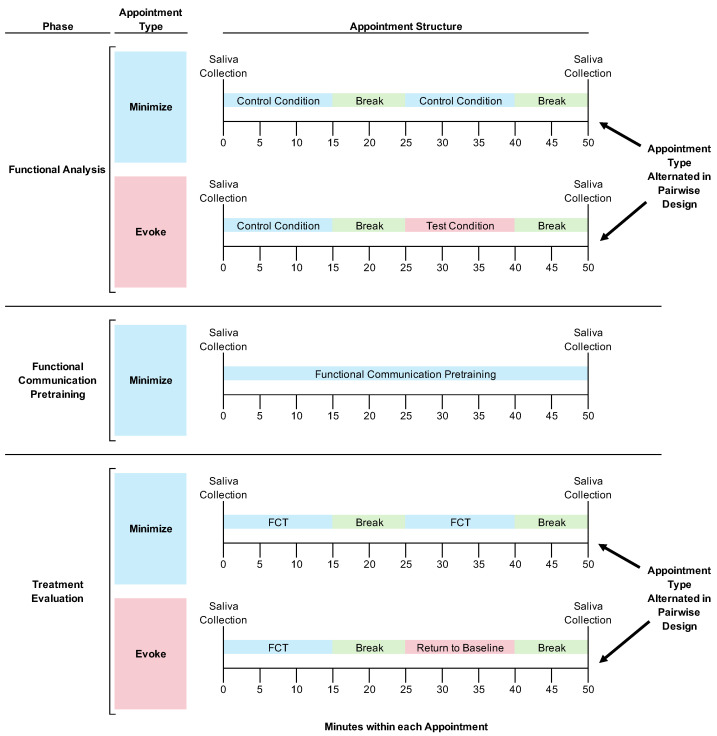
Experimental design and research appointment structure flow chart. ***Note.*** “Minimize” refers to appointments designed to minimize destructive behavior throughout; “Evoke” refers to appointments that included exposure to a condition designed to evoke destructive behavior (e.g., test condition, return to baseline condition). Within the functional analysis and treatment evaluation phases, appointment types were alternated quasi-randomly within in a pairwise design to demonstrate experimental control.

See Figure 1 for the experimental design and general structure of research appointments depicted as a flow chart.

**FA and Treatment Evaluation Appointments.** We told caregivers and teachers to prevent the participants from eating or drinking (except for water) for at least 1 h prior to appointments to avoid skewing their salivary cortisol concentrations (Salimetrics, LLC, 2021). Caregivers were present and observed all procedures (Horace’s caregiver observed the procedures through a one-way observation window; Boris’ caregiver directly implemented the research procedures, as described below).

Each 1 h appointment started with a saliva collection. After the saliva collection, the participants always experienced a 15 min session designed to minimize the likelihood of destructive behavior, which we hypothesized would also minimize the participants’ stress. During the FA, this was a control condition (see below for detailed descriptions of this and other conditions). During the treatment evaluation, this was a session with the treatment (i.e., FCT) in place. After the first 15 min session, we implemented a 10 min break, which always consisted of free access to attention from adults and preferred toys.

After the break, we implemented a second 15 min session. This second session was when we manipulated environmental stimuli such that we could evaluate their effects on salivary cortisol concentrations. Specifically, during some appointments, the second session was designed to evoke destructive behavior (i.e., in the FA, this was the test condition; in the treatment evaluation, this was a return-to-baseline condition that was identical to the FA test condition; both conditions are described in detail below). During other appointments, the second session was designed to minimize destructive behavior (i.e., in the FA this was the control condition; in the treatment evaluation, this was a session with the treatment in place; both conditions are described below). After the second 15 min session, we implemented a 10 min break to allow sufficient time for a participant’s salivary cortisol concentration to increase before we then collected the final saliva sample of the appointment. In other words, we collected the final saliva sample 25 min after the onset of the two different conditions we implemented in the second session of the appointment. We did this because 25 min after stressful stimulus onset is the middle of the 20–30 min timeframe when salivary cortisol concentrations tend to peak (Giacomello et al., 2020).

We started every appointment with a condition designed to minimize destructive behavior and then systematically varied the environmental conditions in the second session of the appointment for two reasons. First, starting the appointment with a putatively low-stress situation was akin to the acclimation period commonly used in other research evaluating the effects of acute stressors on salivary cortisol (Kirschbaum et al., 1993; Goodman et al., 2017). Second, starting appointments with conditions designed to minimize destructive behavior allowed us to implement a pairwise design throughout these assessments. Specifically, we could alternate between conditions that did and did not evoke destructive behavior within a single appointment to demonstrate experimental control over destructive behavior. Notably, we could not begin appointments with a condition that was likely to evoke destructive behavior because if the condition was also stressful and increased the participant’s salivary cortisol concentrations, the concentrations would potentially remain elevated for a prolonged period after the offset of an acute stressor (Kirschbaum et al., 1993; Goodman et al., 2017). Thus, implementing a potentially stressful condition early in the appointment would have posed difficulties for interpreting the effects of (a) this condition after a relatively long delay or (b) any conditions following this initial condition.

In summary, participants had their saliva collected at the beginning and end of every appointment. During certain appointments, participants did not experience any stimuli designed to evoke their destructive behavior. During other appointments, participants experienced a 15 min exposure to stimuli designed to evoke their destructive behavior at a timeframe intended to cause their salivary cortisol to increase in time for the saliva collection at the end of the appointment. Thus, on days without exposure to evocative stimuli, we hypothesized that we would see lower salivary cortisol concentrations. On days with exposure to evocative stimuli shortly before the final saliva collection, we hypothesized that we would see elevated salivary cortisol concentrations.

**Functional Communication Pretraining Appointments.** As with other appointments, we told caregivers and teachers to prevent the participants from eating or drinking (except for water) for at least 1 h prior to appointments, and appointments began and ended with saliva collections. The procedures that occurred within each appointment differed. Specifically, during the entire appointment, we implemented training procedures to establish an independent FCR in both participants’ repertoires (see below for a detailed description of these procedures). There was no alternation between conditions in this phase. We focused on training an independent FCR throughout the entire appointment so that we could rapidly establish FCT as a treatment for clinical purposes.

#### 2.6.2. Preliminary Procedures

Before any experimental procedures occurred, we conducted a routine, clinical interview with each participant’s caregiver and used the information to customize the FA conditions that we would implement with each participant. We supplemented this interview with an informal observation of the patient with their caregiver, who was instructed to put common establishing operations for destructive behavior (e.g., removal of a toy, removal of attention, providing instructions) in place and then respond to their child in the way that they ordinarily would in their home. Both Horace’s and Boris’ caregivers reported that their destructive behavior was evoked by (a) removal of preferred items and (b) requests to complete instructions. Specifically, Horace’s caregiver reported that his destructive behavior occurred most reliably when taking his iPad away and when he had to complete academic tasks. Boris’ caregiver reported that his destructive behavior also occurred most reliably when taking an electronic device away (e.g., smartphone) and when he had to clean his bedroom (e.g., putting away toys, folding laundry).

We also conducted a paired-stimulus preference assessment (similar to the methods described by Fisher et al., 1992) with each participant to identify both participants’ most preferred toys, which we could use during subsequent assessment and treatment procedures. The preference assessments corroborated caregivers’ reports and showed that an iPad was the most preferred item for both participants.

#### 2.6.3. Functional Analysis

Functional analysis is considered the “gold standard” for identifying the specific reasons why a person engages in destructive behavior (Hanley et al., 2003; Melanson and Fahmie, 2023). These procedures involve manipulating environmental variables to evaluate which stimuli (a) evoke behavior and (b) cause the behavior to continue to occur. This allows clinicians to develop treatments that address the specific reasons why each individual may engage in their destructive behavior.

We developed an individualized test and control condition for each participant based on the information we obtained during the clinical interview, observation, and preference assessment. Coincidentally, the results of our preliminary procedures were similar for both participants, so the FA conditions we developed were similar.

**Test Condition.** Before each session, we set up the session room with an iPad and instructional material that would be used throughout the session. For Horace, these were materials to complete receptive identification instructions at a table; for Boris, these were materials similar to what he would encounter while cleaning up his room at home (i.e., toys scattered on the floor to be put away in bins, laundry to fold and put in a basket, paper to be put in a recycling bin).

Prior to each session, both participants received 1 min of free access to an iPad. At the start of the session, the session implementer (i.e., a behavior technician for Horace; a caregiver for Boris) removed access to the iPad and began providing instructions using a systematic prompting procedure (for Horace, verbal–model–physical prompts with 5 s between prompts; for Boris, alternating verbal and model prompts with 5 s between prompts) with the materials in the room. If participants engaged in a behavior targeted for reduction (for Horace, SIB; for Boris, disruptive behavior or negative statements), the participant was given a 30 s break from instructions with access to the iPad. After 30 s, the iPad was removed and instructions were presented. These procedures were repeated until the end of the 15 min session.

As described above, we implemented synthesized contingencies for both participants (see Slaton & Hanley, 2018, for review). We presented multiple establishing operations and consequences for destructive behavior simultaneously to increase the probability that a single condition would evoke destructive behavior and potentially induce stress for the purposes of (a) simplifying our comparison of salivary cortisol concentrations across conditions and (b) minimizing the overall number of saliva samples we would need to analyze to complete the evaluation.

**Control Condition.** Both participants were given noncontingent, continuous access to the iPad and a break from instructions. In other words, participants had free access to the iPad for the entire 15 min session, and no instructions were presented.

We alternated between test and control conditions in a pairwise design until we observed reliable differences in rates of destructive behavior across these conditions, which suggested that some aspect of the manipulated contingency was a function of the participants’ destructive behavior.

#### 2.6.4. Functional Communication Pretraining

We conducted functional communication pretraining in a trial-based format. For Horace, a communication device was present with two FCR buttons on the screen: pressing one button produced a 30 s break from instructions and the other button produced 30 s access to the iPad. For Boris, two communication cards were placed within arm’s reach: exchanging one communication card produced a 30 s break from instructions and the other produced 30 s access to the iPad. For Boris, we increased the reinforcement intervals to 120 s during training trials to fully suppress his destructive behavior and minimize any potential stress associated with the training procedures.

Before each trial, participants had free access to an iPad with no instructions being presented. Each trial began with the implementer (for Horace, a behavior technician; for Boris, his caregiver) removing access to the iPad and beginning to implement the same instructions used during the FA test condition. A second session implementer (i.e., a behavior technician for both Horace and Boris) physically guided a break FCR, which produced a break from instructions, and then physically guided an FCR for the iPad. We progressively increased the delay to prompting FCRs based on the participants engaging in low levels of destructive behavior over consecutive sets of 10 trials. Pretraining ended when participants engaged in both FCRs independently for 80% of trials without engaging in any destructive behavior for two consecutive sets of 10 trials.

#### 2.6.5. Treatment Evaluation

**Functional Communication Training (FCT).** These 15 min sessions were similar to the FA test condition, except destructive behavior no longer produced any changes in the environment (i.e., extinction). Instead, FCRs produced reinforcement similar to functional communication pretraining: break FCRs produced escape from instructions and iPad FCRs produced access to the iPad. For Horace, breaks and access to the iPad were provided for 30 s, whereas these intervals were 120 s for Boris.

**Return to Baseline.** These 15 min sessions were identical to the FA test condition: destructive behaviors produced a break and access to the iPad for 30 s intervals for both participants.

## 3. Results

All results are reported using visual analysis of the data, which is the predominant method for evaluating single-case, behavior-analytic research (Horner et al., 2005; Kazdin, 2011; Kratochwill et al., 2013; Ledford & Gast, 2018).

**Figure 2:**
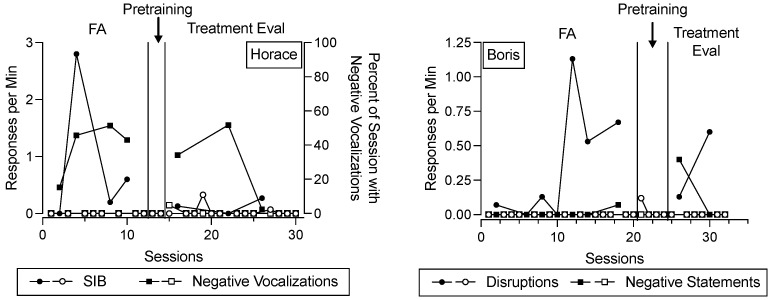
Destructive behavior during functional analysis, functional communication pretraining, and treatment evaluation. ***Note.*** Data points represent the rate of destructive behavior (left *y*-axis) and percent of each session with negative vocalizations (right *y*-axis in the left panel) during sessions throughout the experiment. The shape of the data points corresponds to different topographies of behavior. Data paths with closed data points correspond to conditions designed to evoke destructive behavior across phases (i.e., FA test condition, treatment evaluation return-to-baseline condition), whereas data paths with open data points correspond to conditions designed to minimize destructive behavior across phases (i.e., FA control condition, functional communication pretraining, treatment evaluation FCT condition).

Figure 2 depicts destructive behavior and negative vocalizations/statements across the FA, functional communication pretraining, and treatment evaluation for Horace and Boris. In this figure, data paths with closed data points correspond to conditions designed to evoke destructive behavior across phases, whereas data paths with open data points correspond to conditions designed to minimize destructive behavior across phases. The shape of the data points corresponds to different topographies of behavior (for Horace, open and closed circles represent SIB, open and closed squares represent negative vocalizations; for Boris, open and closed circles represent disruptions, open and closed squares represent negative statements). During the FA and treatment evaluation phases, each data point corresponds to a single 15 min session. During the functional communication pretraining phase, each data point represents the rate or percent of time the behavior occurred throughout an entire appointment. We depict the data in the functional communication pretraining phase in this way because the trial-based format of instruction did not conform to specific session durations, and the training could be paused and resumed at any time. Despite this difference in the level of analysis, we depict these data alongside other behavior data because they are potentially relevant for evaluating in comparison to changes in salivary cortisol concentrations.

For Horace (left panel), SIB occurred at elevated rates in the test condition relative to the control condition during the FA, suggesting that his SIB was maintained by some combination of access to the iPad and/or escape from instructions. His negative vocalizations were also elevated in the test condition relative to the control condition. During functional communication pretraining, SIB and negative vocalizations remained low. During the treatment evaluation, SIB and negative vocalizations tended to occur to a greater extent in the return-to-baseline condition compared to the FCT condition, suggesting that FCT was efficacious for decreasing his SIB (recall that negative vocalizations were not targeted in his FCT intervention).

For Boris (right panel), disruptive behavior and negative statements occurred at elevated rates in the test condition relative to the control condition during the FA, suggesting that disruptive behavior and negative statements were maintained by some combination of access to the iPad and/or escape from instructions. During functional communication pretraining, his disruptive behavior and negative statements decreased and then remained low. During the treatment evaluation, disruptive behavior and negative statements were elevated in the return-to-baseline phase and low in the FCT condition, suggesting that FCT was efficacious for decreasing these behaviors.

Notably, Figure 2 includes more data points than subsequent figures. This is because Figure 2 displays data on a session-by-session basis, and multiple sessions occurred per appointment. Subsequent figures depict data related to salivary cortisol concentrations on an appointment-by-appointment basis.

**Figure 3:**
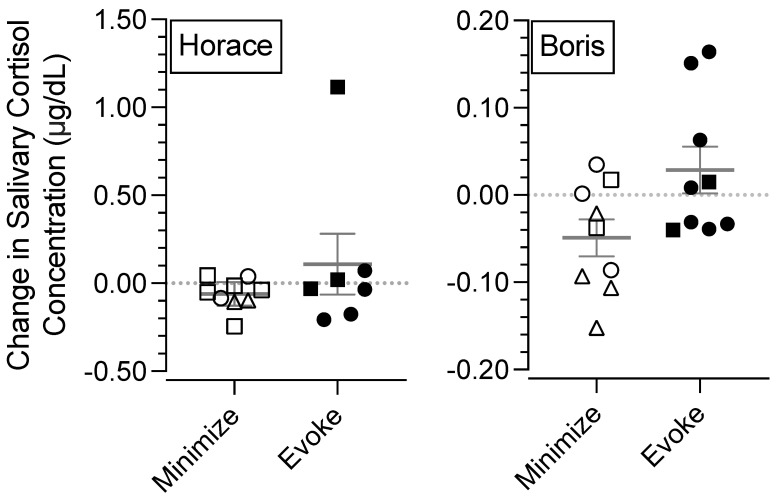
Change in salivary cortisol concentrations. ***Note.*** Symbols correspond to the last 15 min session that occurred prior to the saliva collection at the end of the appointment. Circles, triangles, and squares represent data from the FA, pretraining, and treatment evaluation, respectively. Closed symbols represent conditions designed to evoke destructive behavior. Open symbols represent conditions designed to minimize destructive behavior. Positive values along the *y*-axis indicate increases in salivary cortisol concentration. Negative values indicate decreases in salivary cortisol concentration. Lines represent means, and error bars represent standard error of the mean.

Figure 3 depicts changes in salivary cortisol for Horace (left panel) and Boris (right panel). Similar to Figure 2, closed data points correspond to changes in salivary cortisol concentrations after conditions designed to evoke destructive behavior. In other words, closed data points depict the change in salivary cortisol when the second 15 min session of the appointment (i.e., the session that occurred shortly before the final saliva collection of the day) was designed to evoke destructive behavior (i.e., the FA test condition, the treatment evaluation return-to-baseline condition). Open data points correspond to changes in salivary cortisol concentrations when the entire appointment only included procedures designed to minimize destructive behavior (i.e., the FA control condition, functional communication pretraining, and the treatment evaluation FCT condition). The shape of the data points corresponds to the phase during which the saliva samples were collected: circles, triangles, and squares represent data from the FA, functional communication pretraining, and treatment evaluation, respectively.

Data points that depict changes in salivary cortisol were calculated by subtracting the cortisol concentration obtained from the sample collected at the beginning of an appointment from the concentration obtained from the sample collected at the end of the same appointment. Thus, positive values indicate increases in salivary cortisol concentrations, data points near 0 indicate minimal changes, and negative values indicate decreases in salivary cortisol over the course of the appointment.

For Horace, changes in salivary cortisol concentrations were similarly small regardless of exposure to different conditions. There was a single exception where there was a large increase in salivary cortisol concentration following one of the exposures to the return-to-baseline condition (i.e., a condition designed to evoke destructive behavior).

For Boris, there were two occasions when his salivary cortisol concentration substantially increased after experiencing conditions designed to evoke his destructive behavior. This never occurred after he experienced conditions designed to minimize his destructive behavior—his salivary cortisol either remained similar or decreased after these conditions. Notably, salivary cortisol concentrations generally tend to be high in the morning and decrease throughout the day (Horrocks et al., 1990; Salimetrics, LLC, 2021), so decreases in salivary cortisol concentration also tend to demonstrate the absence of an acute stressful experience. When Boris experienced conditions designed to evoke destructive behavior near the end of his appointments, there were occasions when his salivary cortisol concentrations increased. Recall that previous research shows that increases in salivary cortisol tend to correspond to experiencing acute stressors. Thus, the finding that Boris’ salivary cortisol concentrations increased after being exposed to putative stressors on multiple occasions tends to be consistent with this finding.

**Figure 4:**
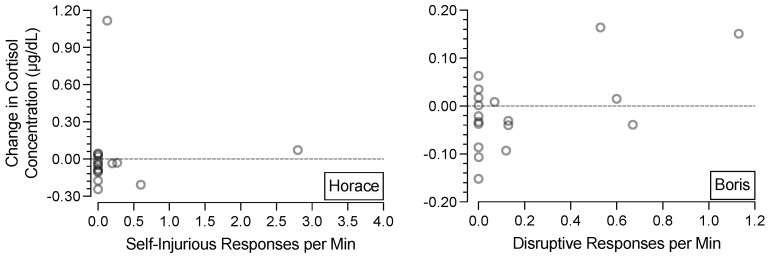
Correlation between destructive behavior and change in cortisol concentration. ***Note.*** Change in salivary cortisol concentration (*y*-axis) as a function of the rate of destructive behavior (*x*-axis) in the second 15-min session of the appointment (i.e., the session preceding the final saliva collection of the appointment).

Figure 4 depicts participants’ changes in salivary cortisol concentrations (*y*-axis) as a function of the rate of their destructive behavior (*x*-axis) in the second 15 min session of the appointment (i.e., the session preceding the final saliva collection of the appointment). For Horace (left panel), there did not appear to be a relation between his SIB and changes in his salivary cortisol. For Boris (right panel), there appeared to be a tendency for changes in salivary cortisol concentrations to increase as the rates of his destructive behavior increased.

**Figure 5:**
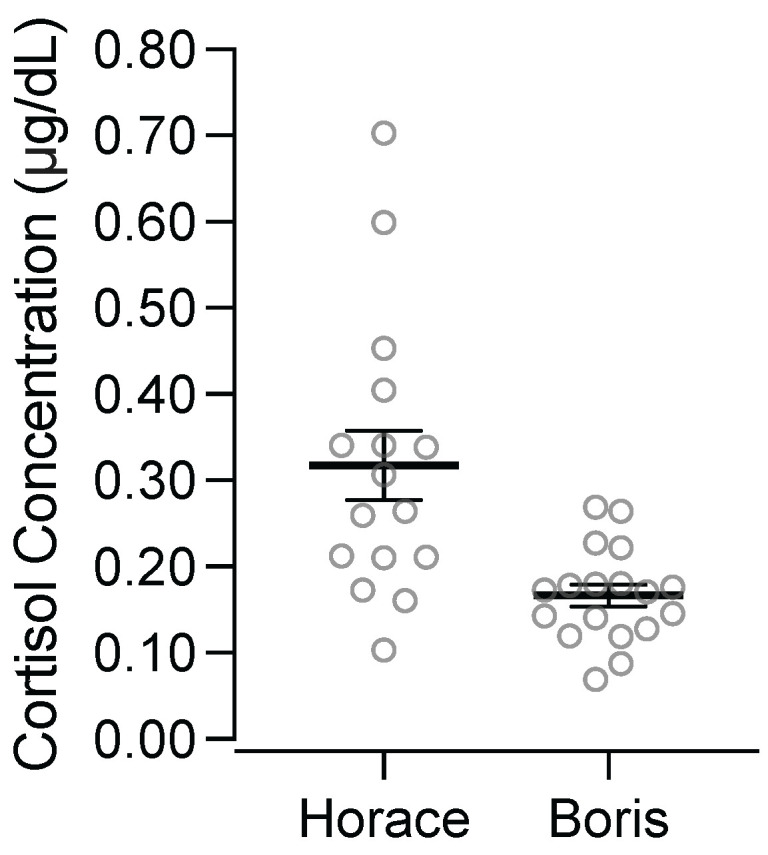
Salivary cortisol concentrations from the first sample of each appointment. ***Note.*** Lines represent means, and error bars represent standard error of the mean.

Figure 5 depicts participants’ salivary cortisol concentrations from the first sample of each appointment. Horace’s salivary cortisol concentration in the first sample collected at each appointment was an average of 0.32 ug/dL (standard deviation = 0.16). Boris’ salivary cortisol concentration in the first sample collected at each appointment was an average of 0.17 ug/dL (standard deviation = 0.05). Notably, Horace’s salivary cortisol concentrations in the first sample of each appointment were substantially higher and more variable than Boris’.

## 4. Discussion

We evaluated two participants’ salivary cortisol concentrations during the assessment and treatment of their destructive behavior. For both participants, we identified (a) specific environmental conditions that reliably evoked their destructive behavior (i.e., removal of preferred items, presentation of instructions), (b) specific environmental conditions that reliably minimized their destructive behavior (i.e., continuous access to preferred items, absence of instructions), and (c) a treatment (i.e., FCT) that minimized destructive behavior during exposures to the environmental stimuli that previously evoked their destructive behavior. For one participant (Boris), exposure to conditions that evoked destructive behavior corresponded with greater increases in salivary cortisol concentrations. For the other participant (Horace), changes in salivary cortisol were small regardless of the conditions he experienced, except for a single instance when he had a large increase in salivary cortisol concentration after an exposure to a condition that also evoked his destructive behavior. Based on these preliminary data, salivary cortisol concentration may be a useful biomarker in the assessment and treatment of destructive behavior; however, more research is needed to more clearly establish its utility for this purpose due to the variability in the data obtained with this small sample of participants.

A potentially important finding from this experiment is the correspondence between Boris’ destructive behavior and the changes in his salivary cortisol concentrations. Figure 3 shows that conditions designed to evoke his destructive behavior tended to correspond with (a) more destructive behavior, (b) more frequent and larger increases in salivary cortisol concentrations, and (c) smaller and fewer decreases in salivary cortisol concentrations compared to conditions designed to minimize his destructive behavior. For example, the three largest increases in salivary cortisol concentrations occurred in conditions that evoked destructive behavior, and conditions that evoked destructive behavior never produced decreases in salivary cortisol that were lower than the mean of the changes in salivary cortisol concentrations associated with conditions that did not evoke destructive behavior. This relation is further illustrated in Figure 4, which shows that the rates of Boris’ destructive behavior tended to correspond with the direction and magnitude of the changes in his salivary cortisol concentrations.

Although this correspondence does not imply that destructive behavior causes stress or vice versa, the correspondence is important because it may help establish a way to assess stress as an outcome measure during the assessment and treatment of destructive behavior for people who are otherwise unable to communicate about their stress. Previous research has firmly established that changes in salivary cortisol concentrations are a reliable biomarker for acute stress (Giacomello et al., 2020), with exposures to acute stressors producing increases in salivary cortisol concentrations. When Boris’ results are considered in relation to this previous research, it suggests that the same environmental stimuli that evoked Boris’ destructive behavior may have induced stress for him. Importantly, in this experiment, we manipulated environmental stimuli (i.e., inaccessibility of an iPad, instructions to complete tasks) that Boris experienced multiple times daily, as evidenced by his referral for the treatment of these behaviors. In this way, Boris likely experienced situations that induced stress frequently throughout his daily routine.

Stress can have widespread effects on a person’s quality of life (e.g., Bishop-Fitzpatrick et al., 2015; McQuaid et al., 2022), so it may be important to target stress as a clinical outcome per se. Boris’ results suggest that salivary cortisol concentrations may be a promising outcome measure that researchers can begin using to assess stress for people who are otherwise unable to communicate about their stress. For example, researchers might begin using salivary cortisol concentration as an outcome measure when refining assessment and treatment procedures to help ensure (a) that participants’ stress is minimized throughout assessment procedures and (b) that treatments do not simply decrease destructive behavior but also decrease people’s stress.

Of course, the promising nature of Boris’ results is tempered substantially by the general lack of correspondence between Horace’s destructive behavior and changes in his salivary cortisol. These results may suggest that the environmental stimuli we manipulated simply did not induce a physiological stress response, and Horace’s SIB may have occurred regardless of a stress response. There are, however, at least two important considerations regarding the absence of correspondence for Horace. First, the one time he had a substantial increase in salivary cortisol concentration occurred after a condition that also reliably evoked his destructive behavior. This could have been due to several possibilities: perhaps these conditions caused stress on certain, but not all, occasions for Horace, or perhaps we need to refine our assessment procedures to produce more consistent results.

A second consideration is that Horace’s baseline salivary cortisol concentrations were high and may have produced a ceiling effect that precluded correspondence between his destructive behavior and changes in his salivary cortisol concentrations. Figure 5 shows that the first sample we collected in each appointment had elevated salivary cortisol concentrations for Horace. As a point of comparison, research on normative salivary cortisol concentrations with children shows that males between the ages 11 and 20 have an average of 0.08 μg/dL at 1 pm, with the fifth percentile = 0.02 and the 95th percentile = 0.26 (Miller et al., 2016). Thus, on average, Horace’s starting concentration (*M* = 0.32) was above the 95th percentile of the sample from Miller et al. Further, Putnam et al. (2015) evaluated diurnal patterns of salivary cortisol concentrations across typically developing children and children with varying levels of ASD, and they found that samples collected near 12 pm had a mean of 0.09 μg/dL (*SD* = 0.03) for typically developing participants, 0.10 μg/dL (*SD* = 0.05) for participants described as having high-functioning ASD, and 0.16 μg/dL (*SD* = 0.05) for participants described as having low-functioning ASD. Clearly, Horace’s average starting concentration was substantially higher than the means from the afternoon saliva samples across these participants with varying diagnoses, even though these saliva samples were collected earlier in the day when concentrations tend to be higher. Thus, Horace’s elevated baseline salivary cortisol concentrations may have limited our ability to detect increases related to the presentation of acute stressors, thereby limiting the possibility of observing correspondence between changes in his salivary cortisol concentration and his destructive behavior. Indeed, this is why standard research practice is to conduct assessments evaluating salivary cortisol concentration reactivity at times of the day when the concentrations tend to be lower (i.e., times late in the day tend to be associated with lower salivary cortisol concentrations; Horrocks et al., 1990; Salimetrics, LLC, 2021). Until salivary cortisol concentration is more firmly established as a viable outcome measure in research on the assessment and treatment of destructive behavior, future researchers might consider conducting screenings for baseline salivary cortisol concentrations for the purpose of excluding participants with high concentrations.

### 4.1. Limitations and Future Directions

Our small sample of participants limits the generalizability of our findings, and future research should evaluate the extent to which our results might be obtained with additional participants. For example, future research with larger numbers of participants could consider evaluating how different participant characteristics or behavioral presentations (e.g., topographies, intensities, and rates of behavior) may relate to salivary cortisol reactivity.

There are potential issues with the feasibility of using salivary cortisol concentrations as a biomarker for stress. For example, due to the diurnal pattern of salivary cortisol concentrations (i.e., high concentrations in the morning and low concentrations in the evening, Horrocks et al., 1990; Salimetrics, LLC, 2021), evaluations using salivary cortisol concentration should be conducted late in the day, which can pose issues for scheduling the procedures. Another factor that may limit feasibility is that participants must cooperate with saliva collection procedures; however, noncooperation with sampling or measurement procedures would also be a potential limitation for using any biomarker as an index for stress (e.g., using heart rate would require cooperation with wearing devices that measure heart rate). Another consideration is that repeated exposures to the same stressor may cause habituation and a decreased physiological stress response regardless of treatment implementation (Grissom & Bhatnagar, 2009). This could make it difficult for researchers to differentiate between the potential effects of habituation and other variables on physiological stress responses when conducting within-subject experiments involving repeated exposure to stressors.

Future research could also consider evaluating other dependent variables related to stress, because there are numerous other biomarkers for stress, such as testosterone, dehydroepiandrosterone-sulfate, 3-methoxy-4-hydroxyphenylglycol, salivary alpha-amylase, secretory immunoglobulin A, and chromogranin A (see Giacomello et al., 2020, for review). A limitation of the aforementioned dependent variables and salivary cortisol is that they require laboratory testing, so they cannot provide real-time information about stress. Although these post hoc measures could help inform research, they may have limited utility for informing clinical decision-making for individual patients.

Conversely, heart rate and galvanic skin response could provide real-time information about stress and can also be assessed noninvasively (Sharma et al., 2016). Notably, McCabe and Greer (2023) evaluated heart rate as a dependent measure during FAs of destructive behavior for four participants and observed no correspondence among heart rate, destructive behavior, or the FA conditions. Their preliminary research suggests that it might be difficult to detect correspondence between environmental stimuli that cause destructive behavior and heart rate, which may suggest that it would also be difficult to detect correspondence between heart rate and other dependent variables (e.g., stress) that may covary with environmental stimuli or destructive behaviors. Correspondence between galvanic skin response and destructive behavior does not seem to have been studied previously and may be a promising dependent variable to evaluate, especially with the increasing commercial availability of more comfortable wearable measurement devices.

Another general area of research would be to explore the effects of several procedural variations. For example, Boris’ caregiver implemented all procedures, whereas a behavior technician implemented procedures for Horace. We observed a tendency toward correspondence between destructive behavior and salivary cortisol concentrations for Boris but not Horace, so it may be the case that the procedures need to closely align with the naturally occurring stressors to more reliably produce a physiological stress response. Similarly, there has been considerable procedural variation in how previous research has used salivary cortisol concentrations to assess stress related to the presentation of acute stressful stimuli (Goodman et al., 2017). For example, different researchers have used different (a) acclimation period durations, (b) conditions during the acclimation period, (c) stressful stimuli, (d) durations of exposure to stressful stimuli, (e) interval lengths between saliva samples, (f) times of day, and (g) participant characteristics. All of these variables can affect the magnitude of changes in salivary cortisol concentrations and the likelihood of detecting these changes (Goodman et al., 2017). We used procedures similar to those used previously; however, future researchers may consider modifying our procedures to enhance the likelihood of detecting changes in salivary cortisol.

### 4.2. Conclusions

This research evaluated correspondence between environmental manipulations, destructive behavior, and salivary cortisol concentrations. We observed correspondence among these variables for one participant. Although we did not observe correspondence for the other participant, there are several explanations for the absence of correspondence that could be addressed in future research (e.g., excluding participants with chronically elevated salivary cortisol concentrations). Given the potential significance of stress as an outcome measure per se and the difficulty associated with assessing this variable with people with limited communication skills, future research should continue to evaluate the viability of using biomarkers as measures of stress, including salivary cortisol concentration. Similar to McCabe and Greer (2023), this preliminary experiment demonstrates a potential methodology for evaluating biological mechanisms associated with behaviors in a way that can facilitate collaboration between behavioral and biological sciences.

## Data Availability

The data presented in this study are available upon request from the corresponding author to ensure participant privacy.

## References

[B1-behavsci-15-00475] Bernstein A. M., Clark S. B., Pattishall A. E., Morris C. R., McCarter A., Muething C. S., Pavlov A. C., Chun T., Call N. A. (2024). The development and acceptability of a comprehensive crisis prevention program for implementation in health care settings. Journal of the American Psychiatric Nurses Association.

[B2-behavsci-15-00475] Bishop-Fitzpatrick L., Mazefsky C. A., Minshew N. J., Eack S. M. (2015). The relationship between stress and social functioning in adults with autism spectrum disorder and without intellectual disability. Autism Research: Official Journal of the International Society for Autism Research.

[B3-behavsci-15-00475] Bullock C. E., Fisher W. W., Hagopian L. P. (2017). Description and validation of a computerized behavioral data program: “BDataPro”. The Behavior Analyst.

[B4-behavsci-15-00475] Campbell J. M. (2003). Efficacy of behavioral interventions for reducing problem behavior in persons with autism: A quantitative synthesis of single-subject research. Research in Developmental Disabilities.

[B5-behavsci-15-00475] Corbett B. A., Mendoza S., Abdullah M., Wegelin J. A., Levine S. (2006). Cortisol circadian rhythms and response to stress in children with autism. Psychoneuroendocrinology.

[B6-behavsci-15-00475] Danese E., Padoan A., Negrini D., Paviati E., De Pastena M., Esposito A., Lippi G., Montagnana M. (2024). Diurnal and day-to-day biological variation of salivary cortisol and cortisone. Clinical Chemistry and Laboratory Medicine.

[B7-behavsci-15-00475] Fisher W., Piazza C. C., Bowman L. G., Hagopian L. P., Owens J. C., Slevin I. (1992). A comparison of two approaches for identifying reinforcers for persons with severe and profound disabilities. Journal of Applied Behavior Analysis.

[B8-behavsci-15-00475] Foley G. M., Baz T. (2021). “Aggression” in young children on the autistic spectrum: The dysregulation–“aggression” hypothesis. Emerging programs for autism spectrum disorder.

[B9-behavsci-15-00475] Giacomello G., Scholten A., Parr M. K. (2020). Current methods for stress marker detection in saliva. Journal of Pharmaceutical and Biomedical Analysis.

[B11-behavsci-15-00475] Ghaemmaghami M., Hanley G. P., Jessel J. (2021). Functional communication training: From efficacy to effectiveness. Journal of Applied Behavior Analysis.

[B10-behavsci-15-00475] Goodman W. K., Janson J., Wolf J. M. (2017). Meta-analytical assessment of the effects of protocol variations on cortisol responses to the Trier Social Stress Test. Psychoneuroendocrinology.

[B12-behavsci-15-00475] Greer B. D., Fisher W. W., Saini V., Owen T. M., Jones J. K. (2016). Functional communication training during reinforcement schedule thinning: An analysis of 25 applications. Journal of Applied Behavior Analysis.

[B13-behavsci-15-00475] Grissom N., Bhatnagar S. (2009). Habituation to repeated stress: Get used to it. Neurobiology of Learning and Memory.

[B14-behavsci-15-00475] Hanley G. P., Iwata B. A., McCord B. E. (2003). Functional analysis of problem behavior: A review. Journal of Applied Behavior Analysis.

[B16-behavsci-15-00475] Hellhammer D. H., Wüst S., Kudielka B. M. (2009). Salivary cortisol as a biomarker in stress research. Psychoneuroendocrinology.

[B15-behavsci-15-00475] Heyvaert M., Saenen L., Campbell J. M., Maes B., Onghena P. (2014). Efficacy of behavioral interventions for reducing problem behavior in persons with autism: An updated quantitative synthesis of single-subject research. Research in Developmental Disabilities.

[B17-behavsci-15-00475] Horner R. H., Carr E. G., Halle J., McGee G., Odom S., Wolery M. (2005). The use of single-subject research to identify evidence-based practice in special education. Exceptional Children.

[B18-behavsci-15-00475] Horrocks P. M., Jones A. F., Ratcliffe W. A., Holder G., White A., Holder R., Ratcliffe J. G., London D. R. (1990). Patterns of ACTH and cortisol pulsatility over twenty-four hours in normal males and females. Clinical Endocrinology.

[B19-behavsci-15-00475] Iwata B. A., Dorsey M. F., Slifer K. J., Bauman K. E., Richman G. S. (1982). Toward a functional analysis of self-injury. Analysis and Intervention in Developmental Disabilities.

[B20-behavsci-15-00475] Iwata B. A., Dorsey M. F., Slifer K. J., Bauman K. E., Richman G. S. (1994). Toward a functional analysis of self-injury. Journal of Applied Behavior Analysis.

[B21-behavsci-15-00475] Kazdin A. E. (2011). Single-case research designs: Methods for clinical and applied settings.

[B23-behavsci-15-00475] Kirschbaum C., Strasburger C. J., Langkrär J. (1993). Attenuated cortisol response to psychological stress but not to CRH or ergometry in young habitual smokers. Pharmacology, Biochemistry, and Behavior.

[B22-behavsci-15-00475] Kranak M. P., Briggs A. M. (2025). Efficiency, Safety, and Dissemination: Considerations for Research and Practice Related to the Practical Functional Assessment. Behavioral Interventions.

[B24-behavsci-15-00475] Kratochwill T. R., Hitchcock J. H., Horner R. H., Levin J. R., Odom S. L., Rindskopf D. M., Shadish W. R. (2013). Single-case intervention research design standards. Remedial and Special Education.

[B25-behavsci-15-00475] Kupferstein H. (2018). Evidence of increased PTSD symptoms in autistics exposed to applied behavior analysis. Advances in Autism.

[B26-behavsci-15-00475] Kurtz P. F., Boelter E. W., Jarmolowicz D. P., Chin M. D., Hagopian L. P. (2011). An analysis of functional communication training as an empirically supported treatment for problem behavior displayed by individuals with intellectual disabilities. Research in Developmental Disabilities.

[B27-behavsci-15-00475] Ledford J. R., Gast D. L. (2018). Single case research methodology: Applications in special education and behavioral sciences.

[B28-behavsci-15-00475] Liu J. J. W., Ein N., Peck K., Huang V., Pruessner J. C., Vickers K. (2017). Sex differences in salivary cortisol reactivity to the Trier Social Stress Test (TSST): A meta-analysis. Psychoneuroendocrinology.

[B29-behavsci-15-00475] McCabe L. H., Greer B. D. (2023). Evaluations of heart rate during functional analyses of destructive behavior. Journal of Applied Behavior Analysis.

[B30-behavsci-15-00475] McQuaid G. A., Weiss C. H., Said A. J., Pelphrey K. A., Lee N. R., Wallace G. L. (2022). Increased perceived stress is negatively associated with activities of daily living and subjective quality of life in younger, middle, and older autistic adults. Autism Research: Official Journal of the International Society for Autism Research.

[B31-behavsci-15-00475] Melanson I. J., Fahmie T. A. (2023). Functional analysis of problem behavior: A 40-year review. Journal of Applied Behavior Analysis.

[B32-behavsci-15-00475] Miller R., Stalder T., Jarczok M., Almeida D. M., Badrick E., Bartels M., Boomsma D. I., Coe C. L., Dekker M. C. J., Donzella B., Fischer J. E., Gunnar M. R., Kumari M., Lederbogen F., Power C., Ryff C. D., Subramanian S. V., Tiemeier H., Watamura S. E., Kirschbaum C. (2016). The CIRCORT database: Reference ranges and seasonal changes in diurnal salivary cortisol derived from a meta-dataset comprised of 15 field studies. Psychoneuroendocrinology.

[B33-behavsci-15-00475] Neuhaus E., Kang V. Y., Kresse A., Corrigan S., Aylward E., Bernier R., Bookheimer S., Dapretto M., Jack A., Jeste S., McPartland J. C., Van Horn J. D., Pelphrey K., Webb S. J., ACE GENDAAR Consortium (2022). Language and aggressive behaviors in male and female youth with autism spectrum disorder. Journal of Autism and Developmental Disorders.

[B34-behavsci-15-00475] Putnam S. K., Lopata C., Thomeer M. L., Volker M. A., Rodgers J. D. (2015). Salivary cortisol levels and diurnal patterns in children with autism spectrum disorder. Journal of Developmental and Physical Disabilities.

[B35-behavsci-15-00475] Rajaraman A., Austin J. L., Gover H. C., Cammilleri A. P., Donnelly D. R., Hanley G. P. (2022). Toward trauma-informed applications of behavior analysis. Journal of Applied Behavior Analysis.

[B36-behavsci-15-00475] Roane H. S., Fisher W. W., Carr J. E. (2016). Applied behavior analysis as treatment for autism spectrum disorder. The Journal of Pediatrics.

[B37-behavsci-15-00475] Rozsa M. (2025). A controversial autism therapy is gaining prominence, but some say it hurts neurodiverse people. *Salon*.

[B38-behavsci-15-00475] Salimetrics, LLC (2021). Expanded range high sensitivity salivary cortisol enzyme immunoassay kit.

[B39-behavsci-15-00475] Salimetrics, LLC (n.d.). Saliva collection training videos: Children’s saliva collection swab device training (SCS).

[B40-behavsci-15-00475] Schlotz W., Kumsta R., Layes I., Entringer S., Jones A., Wüst S. (2008). Covariance between psychological and endocrine responses to pharmacological challenge and psychosocial stress: A question of timing. Psychosomatic Medicine.

[B41-behavsci-15-00475] Sharma M., Kacker S., Sharma M. (2016). A brief introduction and review on galvanic skin response. International Journal of Medical Research Professionals.

[B42-behavsci-15-00475] Slaton J. D., Hanley G. P. (2018). Nature and scope of synthesis in functional analysis and treatment of problem behavior. Journal of Applied Behavior Analysis.

[B43-behavsci-15-00475] Taylor J. L., Corbett B. A. (2014). A review of rhythm and responsiveness of cortisol in individuals with autism spectrum disorders. Psychoneuroendocrinology.

[B44-behavsci-15-00475] van der Linden K., Simons C., van Amelsvoort T., Marcelis M. (2022). Emotional stress, cortisol response, and cortisol rhythm in autism spectrum disorders: A systematic review. Research in Autism Spectrum Disorders.

[B45-behavsci-15-00475] Wolfram M., Bellingrath S., Feuerhahn N., Kudielka B. M. (2013). Cortisol responses to naturalistic and laboratory stress in student teachers: Comparison with a non-stress control day. Stress and Health: Journal of the International Society for the Investigation of Stress.

